# The Correlation between Echocardiographic Findings and QT Interval in Cirrhotic Patients

**Published:** 2014-04-01

**Authors:** Alireza Moaref, Mahmood Zamirian, Maryam Yazdani, Oveis Salehi, Mehrab Sayadi, Kamran Aghasadeghi

**Affiliations:** 1 Cardiovascular Research Center, Shiraz University of Medical Sciences, Shiraz, IR Iran

**Keywords:** Cirrhosis, Electrocardiography, Echocardiography, Long QT

## Abstract

**Background:**

Although many electrocardiographic abnormalities have been reported previously, prolonged QTC interval represented as the most important ECG finding in patients with liver cirrhosis. Echocardiography can detect structural cardiac abnormalities in cirrhotic patients.

**Objectives:**

The present study aimed to determine the correlation between QTC prolongation and echocardiographic findings in end stage liver cirrhosis.

**Methods:**

The present study was conducted on 100 patients selected through convenient sampling. We recruited 80 cirrhotic patients with CHILD score > B or MELD score > 15 from the transplantation ward of Nemazee educational hospital. A complete echocardiographic study, including chamber quantification, a complete flow Doppler, and tissue Doppler analysis, was performed for each patient using a GE vivid 3 system equipped with Tissue Doppler Imaging (TDI). Then, twelve-lead ECG was carried out and QTc interval was calculated in all patients. The data were analyzed using the SPSS statistical software (v. 13) and Pearson’s correlation coefficient. P value < 0.05 was considered statistically significant.

**Results:**

The patients’ age ranged from 20 to 60 years old and 62.5% of them were male. According to the results, the only parameter which was significantly associated with prolonged QTc interval was Left Ventricular End Diastolic Dimension (LVEDD). Additionally, a linear direct relationship was found between corrected QT interval and LVEDD (r = 0.41, P < 0.001).

**Conclusions:**

The current study showed a positive correlation between QTC prolongation as an electerocardiographic finding and LVEDD in echocardiography of the cirrhotic patients. This may indicate a direct relationship between the electrophysiological problems and the severity of volume overload in cirrhotic patients.

## 1. Background

Despite the frequent abnormalities in cardiac structure and function in the patients with end-stage liver disease, decreased afterload because of vasodilatation makes the diagnosis of myocardial dysfunction difficult. The patients with cirrhosis have a large spectrum of circulatory disturbances, including increased Cardiac Output (CO), reduced systemic vascular resistance, and increased vascular compliance (1, 2). In the normal condition, these complications are obscure, but when the cirrhotic patients are challenged by pharmacological or physiological stress, ventricular hypo responsiveness occurs. Although the clinical manifestations of cirrhotic cardiomyopathy may be obscure among the patients, some pathophysiological features, such as increased baseline CO, attenuated systolic contraction and diastolic relaxation, electrophysiological abnormalities, and reduced cardiac response to direct β- adrenergic receptor stimulation, usually develop in these patients (3). These pathophysiological features can be easily diagnosed by using paraclinical assessments. Electrophysiological abnormalities in these patients occur in the absence of any known cardiac disease (4). Many electrocardiographic abnormalities have been reported previously; nevertheless, prolonged QT interval represents the most important electerocardiographic finding in the patients with liver cirrhosis (5, 6). Furthermore, Echocardiography can precisely detect CO changes, systolic contraction, and diastolic relaxation abnormalities. Thus, there might be some relationships between these paraclinical methods which can help to detect cirrhotic cardiomyopathy.

## 2. Objectives

The present study aims to evaluate the correlation between echocardiographic and electrophysiological abnormalities in cirrhotic patients.

## 3. Patients and Methods

### 3.1. Study Population

All the subjects were selected from the transplantation ward of Nemazee educational hospital, a referral center for liver transplantation in the Middle East. This study was approved by the Review Board and Ethics Committee of Shiraz University of Medical Sciences, and written informed consents were obtained from all the participants. At first, 100 patients were selected through convenient sampling and after considering the exclusion criteria, 80 patients (30 females and 50 males) with liver cirrhosis of various etiologies were entered into the study. Liver cirrhosis was determined on the basis of liver biopsy, presence of various laboratory data, or image studies including ultrasonography, Computed Tomography (CT) scan, and endoscopic findings which had been performed several months prior to the final enrollment.

The patients were 20 to 60 years old with documented cirrhosis and CHILD class > B or Model for End-stage Liver Disease score (MELD score) > 15.

On the other hand, the patients with coronary artery disease, significant vavular heart disease (moderate to severe), any history of arrhythmia or taking antiarrhythmic drugs, electrolyte imbalances, and history of taking any medication that could prolong QTc interval were excluded from the study.

### 3.2. Surveillance

Surveillance consisted of a clinical examination performed by an experienced physician, imaging studies, and biopsy performed within one month of surveillance of all enrolled patient by an experienced radiologist or gastroenterologist. In addition, all the necessary lab data including a full panel of blood biochemistry, including blood count, liver function, kidney function, and coagulation, were collected. The imaging studies consisted of Ultrasonography and CT scan which were performed by a professional radiologist. Besides, endoscopic procedures and liver biopsy were carried out by an experienced gastroenterologist. Then, the biopsy specimens were observed and diagnosed by a skilled pathologist. Afterwards, another expert pathologist reviewed all the biopsy specimens that formed the basis for diagnosis of cirrhosis. Whenever possible, all the investigations were performed within the same month by the same specialist. All the investigations were documented and scored.

A complete echocardiographic study, including chamber quantification, a complete flow Doppler, and tissue Doppler analysis, was performed using a vivid 3 system (General electric, made in Norway) equipped with Tissue Doppler Imaging (TDI). The frequency of the transducer was 2.5 MHz.

In lateral decubitus, Para sternal M-mode images were used to measure Left Ventricular (LV) and Left Atrial (LA) size. In the 4 chamber view, mitral inflow velocity was measured by positioning the sample volume on the tip of the mitral valve and peak early diastolic velocity (E), peak late diastolic velocity (A wave), and E / A ratio were measured.

In the apical 4-chamber view, the TDI cursor was placed near the septal border of the mitral annulus, where the mitral annulus moved along the sample volume and early diastolic velocity (E’), late diastolic velocity (A’), systolic velocity, Isovolumic Contraction Time (IVCT), Isovolumuc Relaxation Times (IVRT), and E / E’ were measured by pulse wave Doppler. Ejection Fraction (EF), Tricuspid Annular Plane Systolic Excursion (TAPSE), and peak tricuspid regurgitation velocity were also measured at the apical 4 chamber view. Besides, TR velocity was used to calculate the Pulmonary Artery Pressure (PAP) using the simplified Bernouii equation. Diastolic dysfunction was defined by E’ < 8 m / sec, while systolic dysfunction was defined by EF < 55 or IVCT > 65 msec. Recording and calculation of different cardiac chambers dimensions were performed according to the recommendation of American Society of Echocardiography guidelines.

Twelve-lead ECG was carried out in all the patients with paper speed of 25 mm / s by electrocardiography (Kenz-Cardico 1201, Japan). The QT interval duration was manually calculated from the beginning of the q wave to the end of the T wave in all 12 leads. Moreover, the maximal QT interval duration was measured among these 12 leads. QT intervals were corrected in accordance with the rate using the BAZET formula: QTC = QT / √RR. Accordingly, the adjusted QT of 460 milliseconds or more in women and 440 milliseconds or more in men was considered as prolonged QT interval.

### 3.3. Statistical Analysis

All the statistical analyses were performed using the SPSS statistical software (v. 13). Pearson’s correlation coefficient test was employed to determine the strength of linear relationships between the variables. The data were presented as mean ± SD. Besides, P value < 0.05 was considered as statistically significant.

## 4. Results

**Figure 1.**
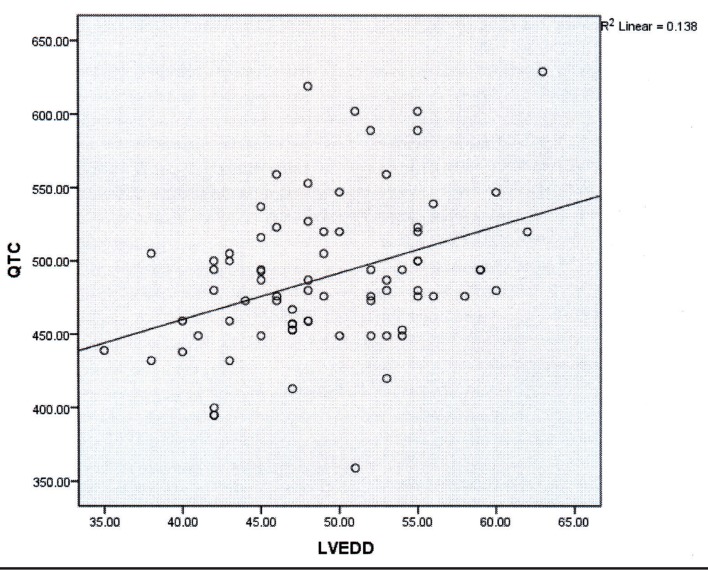
The Correlation between LVEDD and QTc Interval

All the patients were between 20 and 60 years old with the mean age of 41.01 ± 14 years. Besides, the mean QTc interval was 490.02 ± 51 msec. All patients had a normal resting or high normal Left Ventricular (LV) Ejection Fraction (EF). In addition, Pulmonary Acceleration Time (PAT) was greater than 100 msec in all the patients that showed normal pulmonary arterial pressure. Moreover, systolic myocardial velocities at the septal angle of mitral annulus were greater than 0.0 6 m / s in all the patients with the mean value of 0.08 ± 0.01 m / s. Yet, nineteen patients had E’ < 8 cm / s that showed diastolic dysfunction. Also, the mean IVCT was 63.63 ± 7.82 msec and 67% of the patients had IVCT > 65 msec that showed subclinical systolic dysfunction. The mean IVRT was 74.95 ± 9 msec. Moreover, 50% of the patients had E / E’ > 8 that showed increased LV end diastolic pressure at rest. Tricuspid Annular Plane Systolic Excursion (TAPSE) was greater than 20 mm and systolic myocardial velocity at the lateral side of tricuspid annulus was greater than 11 cm / s in all the patients indicating normal RV Function at rest. QTC was prolonged in 65% of the females and 96% of the males. Bivariate analysis showed that the only echocardiographic variable which had a significant relationship with prolonged QTc was LV End Diastolic Dimension (LVEDD) (r = 0.41, P < 0.001). Additionally, a direct linear relationship was found between QTc interval and LVEDD as a marker of volume overload; such a way that the QTc intervals were more prolonged in larger ventricles (Figure 1). In contrast, the parameters associated with systolic and diastolic cardiac function, such as EF, E / A, E / E’, IVCT, IVRT, TAPSE, and PAT, were not associated with prolonged QTC interval. The mean ± SD of all the echocardiographic components has been documented in Table 1. Table 2 shows the correlations between the echocardiographic parameters and QTC interval in details.

**Table 1.** Distribution of Echocardiographic Variables

Echocardiographic Variables	Min	Max	Mean ± SD
LAD (mm)	26.00	46.00	35.66 ± 5.24
LVESD (mm)	20.00	57.00	30.59 ± 5.55
LVEDD (mm)	35.00	63.00	49.01 ± 6.03
SEPTAL S (m / s)	0.05	0.14	0.0863 ±.018
IVCT (ms)	50.00	85.00	63.63 ± 7.82
SEPTAL E´ (m / s)	0.03	0.10	0.085 ± 0.04
E / A	0.6	2.80	1.25 ± 0.39
E / E´	1.00	16.00	8.59 ± 2.64
IVRT (ms)	54.00	100.00	74.95 ± 9.00
TAPSE (mm)	16.00	39.00	24.57 ± 5.03
PAT	100.00	190.00	130.92 ± 20

**Table 2.** The Correlation between the Echocardiographic Parameters and QTc Interval

Echocardiographic Parameters	Pearson Correlation	P value
LAD	0.088	0.447
LVESD	0.134	0.120
LVEDD	0.414	0.001
SEPTAL S	0.228	0.054
IVCT	-0.109	0.341
SEPTAL E´	0.237	0.038
E / A	0.082	0.480
E / E´	0.054	0.644
IVRT	0.186	0.100
TAPSE	0.127	0.279
PAT	-0.107	0.380

## 5. Discussion

Despite the increased basal CO, systemic vascular resistance, arterial pressure, and cardiac response to physiologic or pharmacologic stimuli are decreased in cirrhotic patients. Existence of these components together is the basis of a phenomenon called Cirrhotic Cardiomyopathy (CCM) (7-9). CCM does not show overt heart failure at rest due to decreased afterload because of reduced peripheral vascular resistance (10-12). Although CCM is clinically not so problematic in a resting state, stress conditions, including exercise, infection, drugs, and hemorrhage, and any procedure, such as insertion of Transjugular Intrahepatic Porto-systemic Stent-shunts (TIPS) or liver transplantation, can convert the latent form of CCM to overt heart failure (13, 14). CCM-related heart failure is claimed to be the third leading cause of death after rejection and infection in the transplanted patients (11). Thus, development of a more accurate screening test and assessment criteria for CCM severity estimation seems to be necessary in management of cirrhosis. To achieve this goal, paraclinical assessments, such as electerocardiogram and echocardiography, particularly in combination, can be helpful due to the latent course of CCM (2, 15).

Electrophysiological abnormalities, including prolonged repolarization time and impaired excitation-contraction coupling, have been found in cirrhotic patients (16, 17). Prolongation of QTC interval can be associated with increased risk of certain ventricular arrhythmias (18, 19). The exact mechanism of these electrophysiological abnormalities is still unclear (20). Several hypotheses related to liver dysfunction, alcohol intake, portal hypertension, systemic circulatory disturbances, autonomic dysfunction, porto-systemic shunt, and functional alteration in ion channels have been suggested in clinical studies (21, 22). Other studies have confirmed the reversibility of these electrical phenomena following liver transplantation (23-27). Moreover, some studies have claimed that severity of cirrhosis has some relations with QTC prolongation (28). Although the mechanism of QTC prolongation in cirrhotic patients is not clear yet, there seems to be a strong correlation between QTC prolongation and cirrhosis suggesting QTC interval as the best ECG finding in CCM. Furthermore, some reports have suggested that CCM could be diagnosed with simple echocardiographic diastolic dysfunction indices, such as E / A ratio, even at rest (25). Considering these previously reported findings, the combination of these two paraclinical methods can be beneficial for assessment of CCM in cirrhotic patients.

The present study aimed to determine any relationship between echocardiographic findings and QTC prolongation. The mean QTc interval was 490.02 ± 51 msec. Besides, QTc interval was prolonged in 65% of females and 96% of males, supporting the previous studies (29). Although we found many abnormal echocardiographic components in our population, the only echo index that showed intermediate correlation with QTC interval was LVEDD (r = 0.41, P < 0.001). LVEDD is a parameter which suggests overloaded heart due to increased CO and venous return. Overall, the patients become more overloaded by progression of cirrhosis because of water and salt retention. Additionally, QTc is more prolonged by progress of cirrhosis. Therefore, we can conclude that there is a relationship between overloaded heart and prolonged QTc. Also, overloaded heart may lead to prolongation of the repolarization time by stretching the myofibers. Accordingly, we suggest that volume overload in cirrhotic patients may be a reason for prolongation of QTc.

Left Ventricular Ejection Fraction (LVEF) was normal or supernormal in all the patients in the resting state, which indicates normal or high CO. PAT was also normal in all the patients (PAT > 100 msec), showing normal pulmonary arterial pressure. Besides, TAPSE was greater than 20 mm and systolic myocardial velocity at the lateral side of tricuspid annulus was greater than 0.11 m / s in all the patients. These items showed normal RV systolic function in the resting state. The normal CO in the presence of many cardiac problems may be due to the decreased peripheral vascular resistance (1). In contrast, 19 patients had E’ < 8 that showed diastolic dysfunction. Additionally, the mean IVCT was 63.63 ± 7.82 msec and 67% of the patients had IVCT > 65 msec that showed subclinical systolic dysfunction. Moreover, the mean IVRT was 74.95 ± 9 msec. In 50% of the patients, E / E’ ratio was more than 8 that can suggest increased LV end diastolic pressure at rest. These findings revealed that the study patients had systolic and diastolic myocardial dysfunction, which is consistent with the findings of the previous studies (11).

Considering the relationship between QTc prolongation and overloaded heart, based on the findings of our study, tight volume control in the cirrhotic patients with prolonged QTc is strongly recommended due to the possibility of decompensation after each procedure.

In conclusion, our study showed a positive correlation between QTc prolongation, as an EKG finding, and LVEDD in echocardiography of cirrhotic patients. This may indicate a relationship between the electrophysiological problems and overloaded heart in these patients.

## References

[1] Alqahtani SA, Fouad TR, Lee SS Cirrhotic cardiomyopathy. Seminars in liver disease.

[2] Gaskari SA, Honar H, Lee SS (2006). Therapy insight: Cirrhotic cardiomyopathy.. Nat Clin Pract Gastroenterol Hepatol..

[3] Finucci G, Desideri A, Sacerdoti D, Bolognesi M, Merkel C, Angeli P (1996). Left ventricular diastolic function in liver cirrhosis.. Scand J Gastroenterol..

[4] Bernardi M, Calandra S, Colantoni A, Trevisani F, Raimondo ML, Sica G (1998). Q-T interval prolongation in cirrhosis: prevalence, relationship with severity, and etiology of the disease and possible pathogenetic factors.. Hepatology..

[5] Fourlas CA, Alexopoulou AA (2004). Cirrhotic Cardiomyopathy.. Hellenic J Cardiol..

[6] Møller S, Henriksen JH (2009). Cardiovascular complications of cirrhosis.. Postgraduate medical journal..

[7] Stein LB, Dabezies MA, Silverman M, Brozena SC (1992). Fatal torsade de pointes occurring in a patient receiving intravenous vasopressin and nitroglycerin.. J Clin Gastroenterol..

[8] Zambruni A, Trevisani F, Caraceni P, Bernardi M (2006). Cardiac electrophysiological abnormalities in patients with cirrhosis.. J Hepatol..

[9] Zardi EM, Abbate A, Zardi DM, Dobrina A, Margiotta D, Van Tassell BW (2010). Cirrhotic cardiomyopathy.. J Am Coll Cardiol..

[10] Donovan CL, Marcovitz PA, Punch JD, Bach DS, Brown KA, Lucey MR (1996). Two-dimensional and dobutamine stress echocardiography in the preoperative assessment of patients with end-stage liver disease prior to orthotopic liver transplantation.. Transplantation..

[11] Mikulic E, Munoz C, Puntoni LE, Lebrec D (1983). Hemodynamic effects of dobutamine in patients with alcoholic cirrhosis.. Clin Pharmacol Ther..

[12] Moller S, Henriksen JH (2002). Cirrhotic cardiomyopathy: a pathophysiological review of circulatory dysfunction in liver disease.. Heart..

[13] Baik SK, Fouad TR, Lee SS (2007). Cirrhotic cardiomyopathy.. Orphanet J Rare Dis..

[14] Bernardi M, Rubboli A, Trevisani F, Cancellieri C, Ligabue A, Baraldini M (1991). Reduced cardiovascular responsiveness to exercise-induced sympathoadrenergic stimulation in patients with cirrhosis.. Journal of hepatology..

[15] Wong F, Girgrah N, Graba J, Allidina Y, Liu P, Blendis L (2001). The cardiac response to exercise in cirrhosis.. Gut..

[16] Appleton CP, Hurst RT (2008). Reducing coronary artery disease events in liver transplant patients: moving toward identifying the vulnerable patient.. Liver Transpl..

[17] Ma Z, Lee SS (1996). Cirrhotic cardiomyopathy: getting to the heart of the matter.. Hepatology..

[18] Cazzaniga M, Salerno F, Pagnozzi G, Dionigi E, Visentin S, Cirello I (2007). Diastolic dysfunction is associated with poor survival in patients with cirrhosis with transjugular intrahepatic portosystemic shunt.. Gut..

[19] De BK, Majumdar D, Das D, Biswas PK, Mandal SK, Ray S (2003). Cardiac dysfunction in portal hypertension among patients with cirrhosis and non-cirrhotic portal fibrosis.. Journal of hepatology..

[20] Therapondos G, Flapan AD, Dollinger MM, Garden OJ, Plevris JN, Hayes PC (2002). Cardiac function after orthotopic liver transplantation and the effects of immunosuppression: a prospective randomized trial comparing cyclosporin (Neoral) and tacrolimus.. Liver Transpl..

[21] Pozzi M, Redaelli E, Ratti L, Poli G, Guidi C, Milanese M (2005). Time-course of diastolic dysfunction in different stages of chronic HCV related liver diseases.. Minerva Gastroenterol Dietol..

[22] Valeriano V, Funaro S, Lionetti R, Riggio O, Pulcinelli G, Fiore P (2000). Modification of cardiac function in cirrhotic patients with and without ascites.. Am J Gastroenterol..

[23] Adigun AQ, Pinto AG, Flockhart DA, Gorski JC, Li L, Hall SD (2005). Effect of cirrhosis and liver transplantation on the gender difference in QT interval.. Am J Cardiol..

[24] Campbell R, Day C, James O, Butler T (1993). QT prolongation and sudden cardiac death in patients with alcoholic liver disease.. The Lancet..

[25] Carey EJ, Douglas DD (2005). Effects of orthotopic liver transplantation on the corrected QT interval in patients with end-stage liver disease.. Digestive diseases and sciences..

[26] Genovesi S, Prata Pizzala DM, Pozzi M, Ratti L, Milanese M, Pieruzzi F (2009). QT interval prolongation and decreased heart rate variability in cirrhotic patients: relevance of hepatic venous pressure gradient and serum calcium.. Clin Sci (Lond)..

[27] Lazzeri C, La Villa G, Laffi G, Vecchiarino S, Gambilonghi F, Gentilini P (1997). Autonomic regulation of heart rate and QT interval in nonalcoholic cirrhosis with ascites.. Digestion..

[28] Braverman AC, Steiner MA, Picus D, White H (1995). High-output congestive heart failure following transjugular intrahepatic portal-systemic shunting.. Chest..

[29] Henriksen JH, Moller S, Ring-Larsen H, Christensen NJ (1998). The sympathetic nervous system in liver disease.. J Hepatol..

